# Supplementary material for: Structural and Biochemical Characterization of a Dye-Decolorizing Peroxidase from Dictyostelium discoideum
Source: Int J Mol Sci. 2021 Jun 10;22(12):6265. doi: 10.3390/ijms22126265 (PMC8230527; doi:10.3390/ijms22126265)
Supplement: Supplementary file 1 [file ijms-22-06265-s001.zip › ijms-1234164-supplementary.pdf]

Supplementary information for

Structural and Biochemical Characterization of a Dye-Decolorizing Peroxidase from *Dictyostelium discoideum*

Amrita Rai [1,2], Johann P. Klare [3], Patrick Y. A. Reinke [1,4,5], Felix Englmaier [6,7], Jörg Fohrer [7,8], Roman Fedorov [4], Manuel H. Taft [1], Igor Chizhov [1,4], Ute Curth [1,4], Oliver Plettenburg [6,7] and Dietmar J. Manstein [1,4,9,*]

1 Institute for Biophysical Chemistry, Hannover Medical School, Fritz Hartmann Centre for Medical Research Carl Neuberg Str. 1, D-30625 Hannover, Germany; Amrita.Rai@mpi-dortmund.mpg.de (A.R.); patrick.reinke@desy.de (P.Y.A.R.); Taft.Manuel@mh-hannover.de (M.H.T.); chizhov.igor@mh-hannover.de (I.C.); curth.ute@mh-hannover.de (U.C.)
2 Department of Structural Biochemistry, Max Planck Institute of Molecular Physiology, D-44227 Dortmund, Germany
3 Department of Physics, University of Osnabrueck, Barbarastrasse 7, D-49076 Osnabrück, Germany; jklare@uni-osnabrueck.de
4 Division for Structural Biochemistry, Hannover Medical School, Carl Neuberg Str. 1, D-30625 Hannover, Germany; Fedorov.Roman@mh-hannover.de
5 Center for Free-Electron Laser Science, German Electron Synchrotron (DESY), Notkestr. 85, D-22607 Hamburg, Germany
6 Institute of Medicinal Chemistry, Helmholtz Zentrum München (GmbH), German Research Center for Environmental Health, Ingolstädter Landstraße 1, D-85764 Neuherberg, Germany; felix.englmaier@helmholtz-muenchen.de (F.E.); oliver.plettenburg@oci.uni-hannover.de (O.P.)
7 Center of Biomolecular Drug Research (BMWZ), Institute of Organic Chemistry, Leibniz University Hannover, Schneiderberg 1b, D-30167 Hannover, Germany; joerg.fohrer@tu-darmstadt.de
8 Department of Chemistry, Clemens-Schöpf-Institute of Organic Chemistry and Biochemistry, Darmstadt Technical University, Alarich-Weiss-Strasse 4, D-64287 Darmstadt, Germany
9 Department of Chemistry, Clemens-Schöpf-Institute of Organic Chemistry and Biochemistry, Darmstadt Technical University, D-30625 Hannover, Germany
* Correspondence: Manstein.Dietmar@MH-Hannover.de; Tel.: +49-511-5323700

```
Dictyostelium_DyPA      ------------------------------------------------------------
YfeX                    ------------------------------------------------------------
TyrA                    ------------------------------------------------------------
BtDyP                   ------------------------------------------------------------
RjDypB                  ------------------------------------------------------------
AauDyPI                 MRLSPVFVALLSGLLAADLGLARSVAPRVADSPAAVTGTRKTSLLKNVAGLPPVPSAAQV
BadDyP                  MRLS-LFVVSVAVLVGS--------SSHVNAAKLGARQTRTTPLLTNFPGQAPLPTLTQH

Dictyostelium_DyPA      ---------------MAQSTVLPMHCLYGIFLEGNLKIQK---------NDQEGLKKFKD
YfeX                    -------------MSQVQSGILPEHCRAAIWIEANVKG---------------EVDALRA
TyrA                    --------MDIQNMPREQLGVCAEGNLHSVYLMFNANDN--------------VESQLRP
BtDyP                   -----MNPFQNSFGGHIPQDVAGKQGENVIFIVYNLTDS------------PDTVDKVKD
RjDypB                  --------MPGPVARLAPQAVLTPPSAASLFLVLVAGDS------------DDDRATVCD
AauDyPI                 AATS------LNTDDIQGDILVGMHKQKQLFYFFAINDPATFKTHLASDIAP-VVASVTQ
BadDyP                  TTESGANDTILPLNNIQGDILVGMKKQKERFVFFQVNDATSFKTALKTYVPERITSAAIL
                                            :         :

Dictyostelium_DyPA      NIKKFTLELDEIDKISPQSRIGGAICFSSDIWDTVTKKISKPKELKSVNTLSSY--MPGT
YfeX                    ASKTFADKLATFEAKFPDAHLGAVVAFGNNTWRALSGGVG-AEELKDFPGYGKG--LAPT
TyrA                    CIANVAQYIYELTDQYSDSAFNGFVAIGANYWDSLYPESR-PEMLKPFPAMQEGNREAPA
BtDyP                   VCANFSAMIRSMRNRFPDMQFSCTMGFGADAWTRLFPDKGKPKELSTFSEIKGEKYTAVS
RjDypB                  VISGIDGPLKAVGFRELAGSLSCVVGVGAQFWDRVSAS-SKPAHLHPFVPLSGPVHSAPS
AauDyPI                 LSNVATQPLVALNIAFSNTGL-LALGVTDNLGDSLFANGQAKDATSFKESTSSWVPQFAG
BadDyP                  ISDPSQQPLAFVNLGFSNTGL-QALGITDDLGDAQFPDGQFADAANLGDDLSQWVAPFTG
                                :  .        :   : . :
                                                                             GXXDG
Dictyostelium_DyPA      SQRDILIHIISDRMDTCFKLAQDTMRNFGEDQLDIKQEIHGFRRVE--ERDLTDFIDGTE
YfeX                    TQFDVLIHILSLRHDVNFSVAQAAMEAFG-DCIEVKEEIHGFRWVE--ERDLSGFVDGTE
TyrA                    IEYDLFVHLRCDRYDILHLVANEISQMF-EDLVELVEEERGFRFMD--SRDLTGFVDGTE
BtDyP                   TPGDLLFHIRAKQMGLCFEFASILDEKLKG-AVVSVDETHGFRYMD--GKAIIGFVDGTE
RjDypB                  TPGDLLFHIKAARKDLCFELGRQIVSALGS-AATVVDEVHGFRYFD--SRDLLGFVDGTE
AauDyPI                 TGIHGVIILASDTTDLIDQQVASIESTFGSSISKLYSLSASIRPGNEAGHEMFGFLDGIA
BadDyP                  TTIHGVFLIGSDQDDFLDQFTDDISSTFGSSITQVQALSGSARPGDQAGHEHFGFLDGIS
                           . .. : .   .            :            . *  :   :   .*:**

Dictyostelium_DyPA      NP----------DGDELRTQYGLVAAGQP--------NEFGSYVFTQRYVHNLKKWYPEP
YfeX                    NP----------AGEETRREVAVIKDG-V--------DAGGSYVFVQRWEHNLKQLNRMS
TyrA                    NP----------KGRHRQEVALVGSEDPE--------FKGGSYIHVQKYAHNLSKWHRLP
BtDyP                   NP---------AVDENPYHFAVIGEEDAD--------FAGGSYVFVQKYIHDMVAWNALP
RjDypB                  NP----------TDDDAADSALIGDEDPD--------FRGGSYVIVQKYLHDMSAWNTLS
AauDyPI                 QPAINGFN-TPLPGQNIVDAGVIITGATNDPITRPSWAVGGSFLAFRQLEQLVPEFNKYL
BadDyP                  QPSVTGWETTVFPGQAVVPPGIILTGRDGDTGTRPSWALDGSFMAFRHFQQKVPEFNAYT
                        :*           .        :                 **::  ::  : :

Dictyostelium_DyPA      LSVQQ-------------------DTVGRTKK----------DSIEIPRDKR--------
YfeX                    VHDQE-------------------MVIGRTKE----------ANEEIDGDER--------
TyrA                    LKKQE-------------------DIIGRTKQ----------DNIEYESEDK--------
BtDyP                   VEQQE-------------------KVIGRHKF----------NDVELSDEEK--------
RjDypB                  TEEQE-------------------RVIGRTKL----------ENVELDDDAQ--------
AauDyPI                 LDNAP----AGSGSLQARADLLGARMVGRWKSGAPIDLTPTADDPALGADAQRNNNFTYS
BadDyP                  LANAIPANSAGNLTQQEGAEFLGARMFGRWKSGAPIDLAPTADDPALGADPQRNNNFDYS
                                          .** *            .     : :

Dictyostelium_DyPA      -------------PITSHVSRTDLS-ENG----------KDLKIVRQSLPYG--------
YfeX                    -------------PETSHLTRVDLK-EDG----------KGLKIVRQSLPYG--------
TyrA                    -------------PLTSHIKRVNLKDENG----------KSIEILRQSMPYG--------
BtDyP                   -------------PGNAHNAVTNIGDD--------------LKIVRANMPFA--------
RjDypB                  -------------PSNSHVTLNTIVDDDG----------VEHDILRDNMAFG--------
AauDyPI                 HAGFDLGSDQSHCPFSAHIRKTRPRADLGGSLTPPNLSAGANSIMRSGIPYGPEVTSAES
BadDyP                  ----DTLTDETRCPFGAHVRKTNPRQDLGG-------PVDTFHAMRSSIPYGPETSDAEL
                                     *  :*        :                 :* .:.:.

Dictyostelium_DyPA      --QIT-GEKGLMFIAYACSLHNIEKQLQS-MFGQL-------------------------
YfeX                    --TAS-GTHGLYFCAYCARLHNIEQQLLS-MFGDT-------------------------
TyrA                    --SLK--EQGLMFISTCRTPDHFEKMLHSMVFGDG-------------------------
BtDyP                   --NTSKGEYGTYFIGYASTFSTTRRMLENMFIGSP-------------------------
RjDypB                  --SLGEAEYGTYFIGYAKDPAVTELMLRRMFLGEP-------------------------
AauDyPI                 ASNTTTQERGLAFVAYQAQLSQGFHFLQQTWADNANFPPGKTPATVGLDPIIGQNNGQPR
BadDyP                  ASGVTAQDRGLLFVEYQSIIGNGFRFQQINWANNANFPFSK-PITPGIEPIIGQTT--PR
                                 *  *                   ..

Dictyostelium_DyPA      ---------DGKHDLLLKYTTPVTGSFYFAPSKKELLEL---------------------
YfeX                    ---------DGKRDAMLRFTKPVTGGYYFAPSLDKLMAL---------------------
TyrA                    ---------AGNHDHLMHFTSALTGSSFFAPSLDFLMQFDN-------------------
BtDyP                   ---------AGNTDRLLDFSTAITGTLFFVPSYDLLGELGE-------------------
RjDypB                  ---------PGNYDRVLDFSTAATGTLFFVPSRDVLESLGDEPAGAESAPEDPVEPAAAG
AauDyPI                 VVNGLLPSNSSASLSIPQFVVSHGGEYFFSPPISAIGGRLSA------------------
BadDyP                  TVGGLDPLNQNETFTVPLFVIPKGGEYFFLPSISALTATIAA------------------
                                  .    :  :  .  *  :* *. . :

Dictyostelium_DyPA      ----------------
YfeX                    ----------------
TyrA                    ----------------
BtDyP                   ----------------
RjDypB                  PYDLSLKIGGLKGVSQ
AauDyPI                 ----------------
BadDyP                  ----------------
```

* - Fully conserved residue
: - Conservation of strong groups
. - Conservation of weak groups

Figure S1: Sequence alignment of *Dictyostelium* DyPA with different bacterial and fungal DyPs. The conserved G/DXXDG motif is shown in a black box. *Dictyostelium discoideum* DyPA (UniProt ID: Q556V8), *Escherichia coli* (strain K12) YfeX (UniProt ID: P76536), *Shewanella oneidensis* (strain MR-1) TyrA (UniProt ID: Q8EIU4), *Bacteroides*

thetaiotaomicron VPI-5482 *Bt*DyP (UniProt ID: Q8A8E8), *Rhodococcus jostii* RHA1 *Rj*DyPB (UniProt ID: Q0SE24), *Auricularia auricula-judae Aau*DyPI (UniProt ID: I2DBY1), *Bjerkandera adusta Bad*DyP (UniProt ID: Q8WZK8)

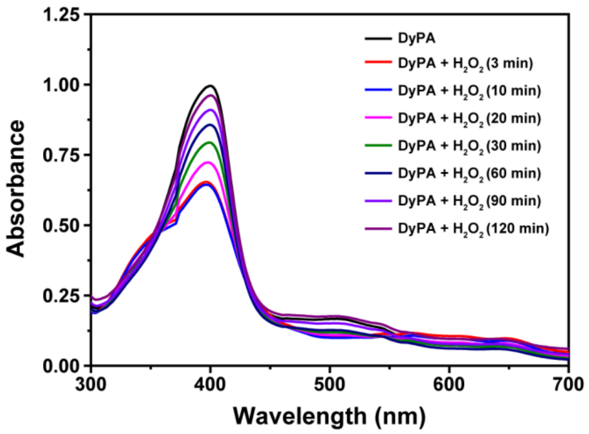

Figure S2: UV-visible absorption spectra of *Dictyostelium* DyPA in the presence of H_2O_2. Spectra of 10 μM *Dictyostelium* DyPA in the absence (black) or in the presence (red to magenta) of 10 μM H_2O_2 at different time points. Measurements performed in buffer containing 50 mM Tris pH 8.0, 150 mM NaCl at 23°C.

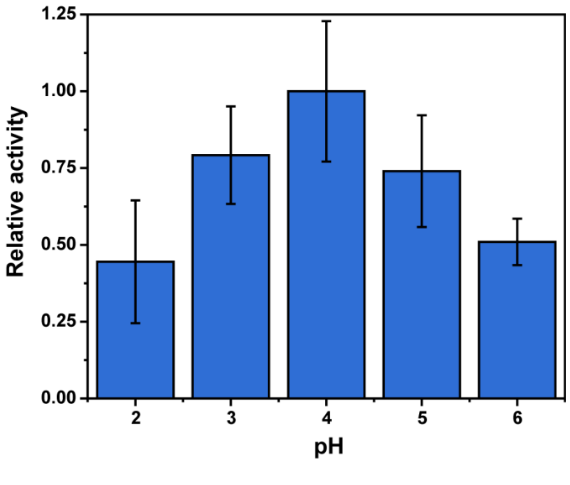

Figure S3: Optimum pH for the oxidation of veratryl alcohol by *Dictyostelium* DyPA. Assays were performed in 100 μl of 50 mM sodium acetate (pH 2.0 – 6.0) and 150 mM NaCl at 25 °C, containing 10 mM VA and 4 μM *Dictyostelium* DyPA. Reactions were initiated by the addition of 1 mM H_2O_2 and monitored at 310 nm ($VA\varepsilon_{310}$= 9.3 mM^{-1} cm^{-1}). Data are average values of 3–6 independent measurements; bars represent the standard deviation.

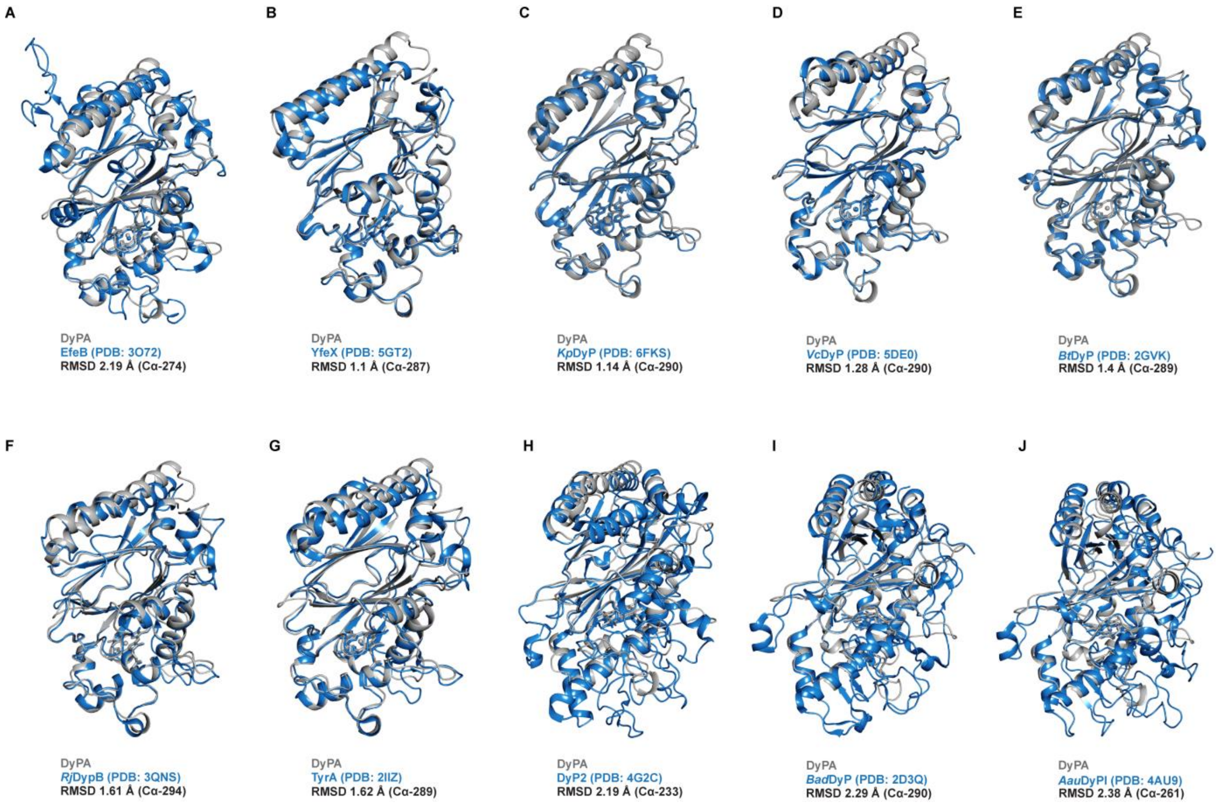

Figure S4. Structural comparison of *Dictyosteliu*m DyPA with related bacterial and fungal dye decolorizing peroxidases. Structural alignment of *Dictyostelium* DyPA (grey) with **(A)** *Escherichia coli* O157 EfeB (blue; class A). **(B)** *Escherichia coli O157* YfeX (blue; class B). **(C)** *Klebsiella pneumoniae Kp*DyP (blue; class B). **(D)** *Vibrio cholerae Vc*DyP (blue; class B). **(E)** *Bacteroides thetaiotaomicron* VPI-5482 *Bt*DyP (blue; class B). **(F)** *Rhodococcus jostii RHA1 Rj*DypB (blue; class B). **(G)** *Shewanella oneidensis* TyrA (blue; class B). **(H)** *Amycolatopsis sp.* 75iv2 DyP2 (blue; class C). **(I)** *Bjerkandera adust*a *Bad*DyP (blue; class D). **(J)** *Auricularia auricula-judae Aau*DyPI (blue; class D).

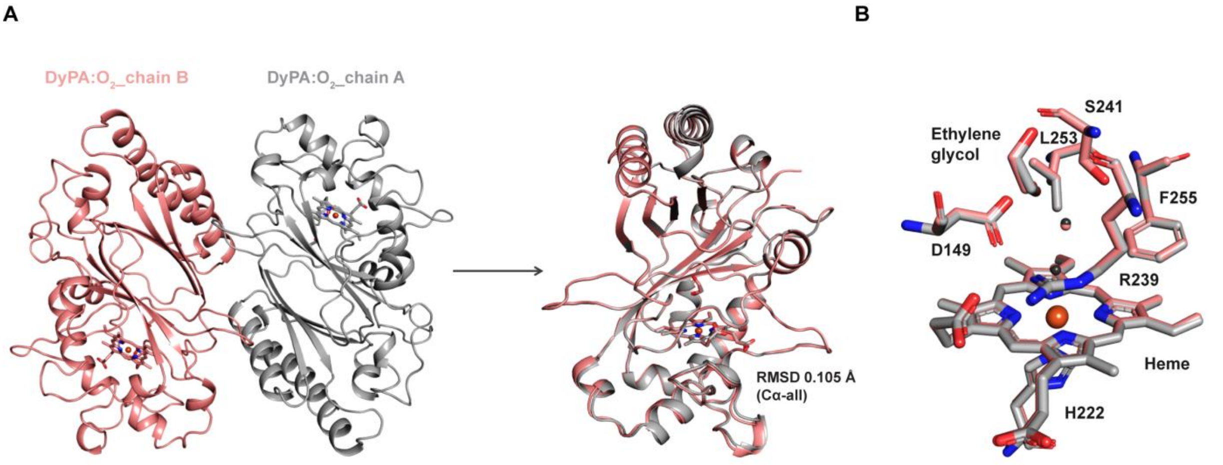

Figure S5: Structure of the *Dictyostelium* DyPA:O_2 complex. **(A)** The asymmetric unit contains two nearly identical copies of DyPA, as shown by the overlay of both structural models. **(B)** Heme microenvironment of the *Dictyostelium* DyPA:O_2 complex. Grey/salmon pink spheres show the position of the oxygen atoms in the *Dictyostelium* DyPA:O_2 complex structure.

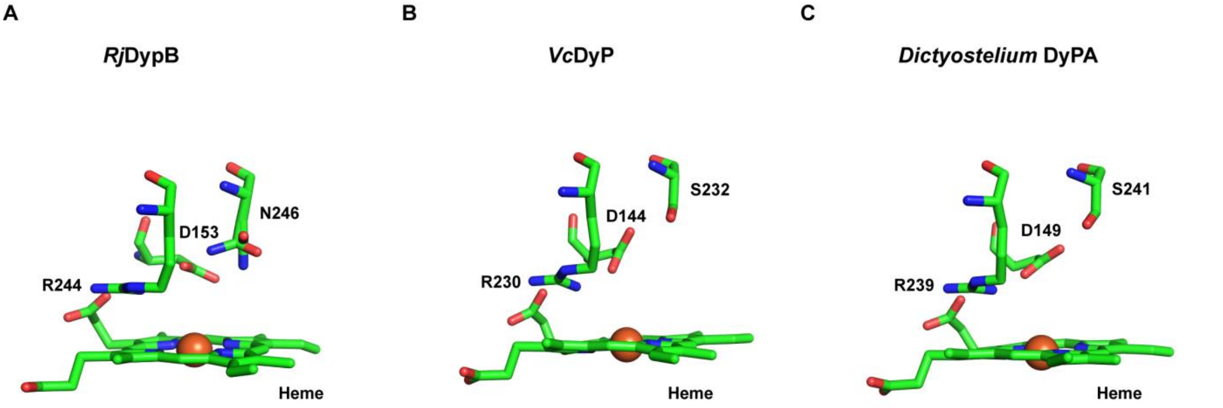

Figure S6. Heme microenvironment. **(A)** *Rj*DypB (PDB: 3QNS), **(B)** *Vc*DyP (PDB: 5DE0) and **(C)** *Dictyostelium* DyPA.

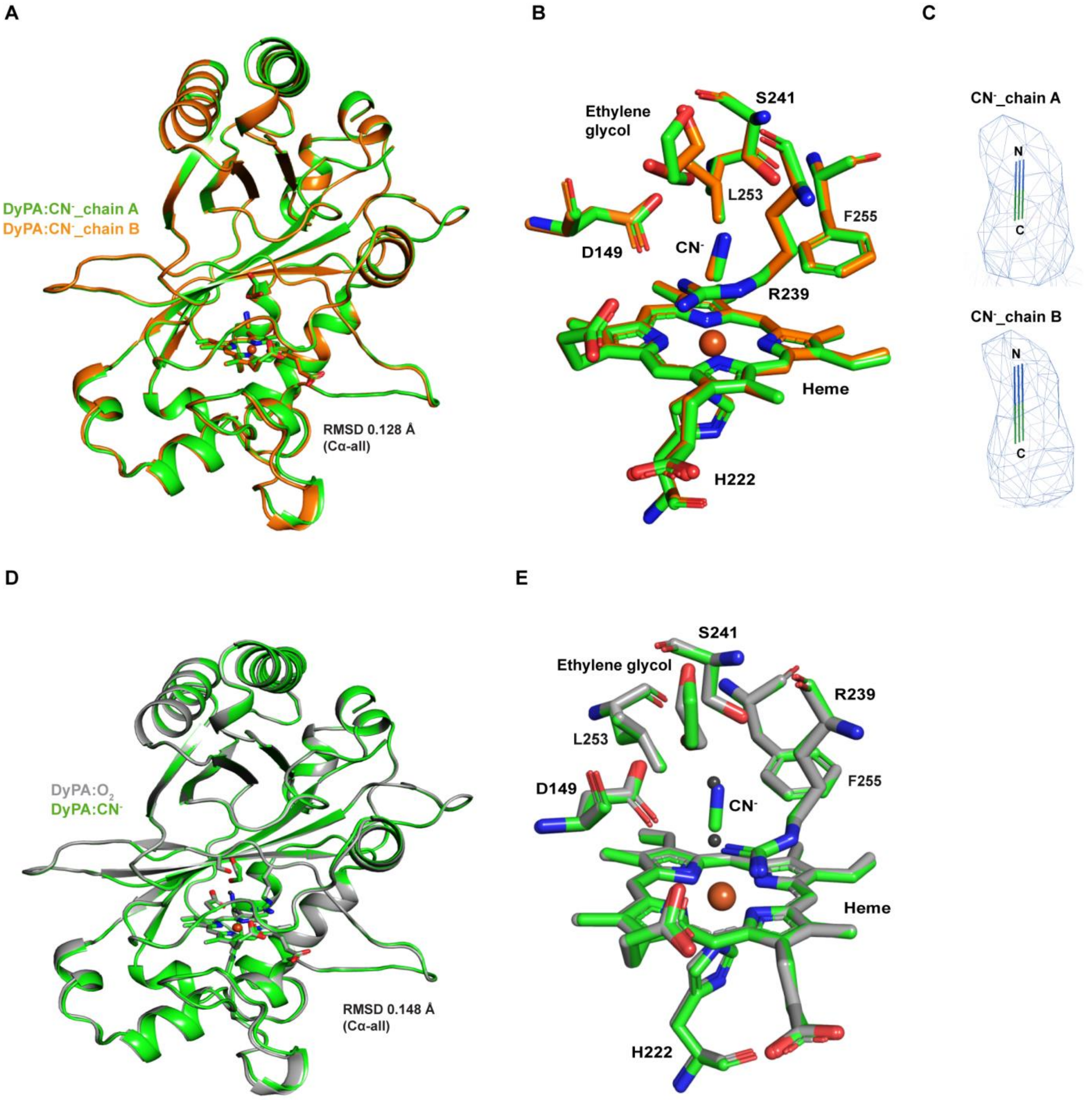

Figure S7: Structure of *Dictyostelium* DyPA:CN^- complex. **(A)** Structural alignment of both copies of the *Dictyostelium* DyPA:CN^- complex in the asymmetric unit reveals that both copies are nearly identical. **(B)** Heme microenvironment of the *Dictyostelium* DyPA:CN^-. (**C)** OMIT electron density maps of CN^- moieties, contoured at 1.5σ. **(D)** Structural alignment of single monomers for the *Dictyostelium* DyPA:O_2 complex structure (grey) and *Dictyostelium* DyPA:CN^- complex (green). **(E)** Overlay of the heme microenvironment of the *Dictyostelium* DyPA CN^- (green) and activated O_2 (grey) complexes. The atoms of the activated oxygen molecule are shown as grey spheres and iron atom of the heme is shown as a red sphere, in the *Dictyostelium* DyPA:O_2 complex structure.

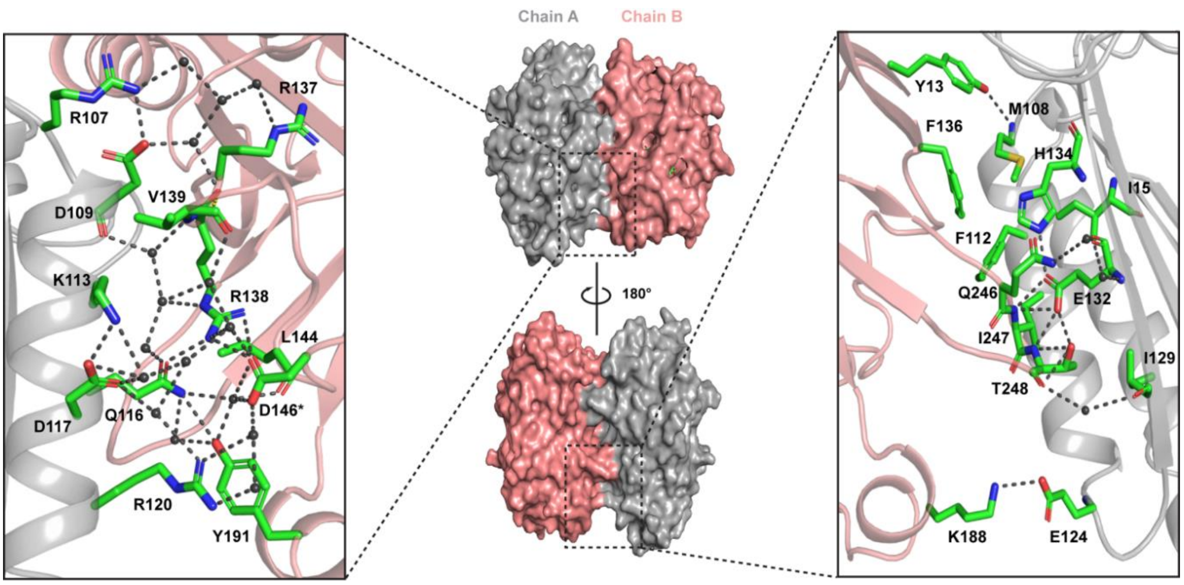

Figure S8: *Dictyostelium* DyPA:O_2 complex dimer interface. The individual *Dictyostelium* DyPA monomers are shown in grey and salmon pink. Surface and cartoon representations of *Dictyostelium* DyPA dimer. Selected amino acids are colored in the atomic color scheme. Water molecules are shown as grey spheres. Key non-covalent bonds are represented as grey dashed lines; the bond length cutoff is 3.5 Å.

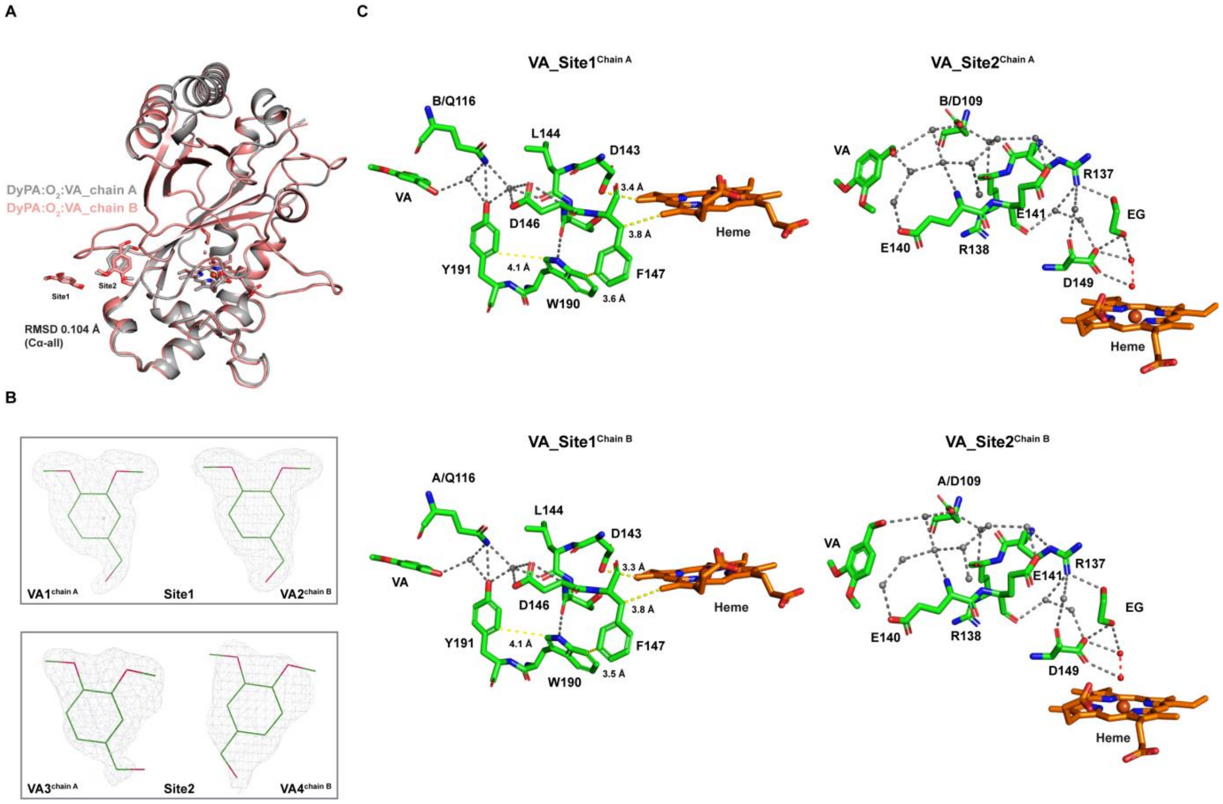

Figure S9: Structure of the *Dictyostelium* DyPA:O2:VA complex. **(A)** Structure of *Dictyostelium* DyPA:O2:VA complex. (A) Structural alignment of both copies of the *Dictyostelium* DyPA:O2:VA complex in the asymmetric unit reveals that both copies are nearly identical (Cα-RMSD of 0.105 Å). **(B)** OMIT electron density maps for bound VA molecules, contoured at 1σ. **(C)** Proposed long-range electron transfer pathways from veratryl alcohol binding sites to the heme moiety of *Dictyostelium* DyPA. The atoms of the activated oxygen molecules are shown as red spheres and the water molecules are shown as grey spheres. Hydrogen bonds are represented as grey dashed lines.

Table S1. Data-collection and refinement statistics
(values in parentheses are for the outer shell).

	**DyPA:O_2**	**DyPA:CN^-**	**DyPA:O_2:VA**
**Data collection#**			
X-Ray Source	ID29, ESRF Grenoble	ID29, ESRF Grenoble	ID23-1, ESRF Grenoble
Wavelength (Å)	0.91985	0.91985	0.910003
Crystal-to-detector distance (mm)	313.233	288.91	318
Exposure time per image (s)	0.0375	0.0375	0.0375
No. of images	1200	1200	3000
Oscillation range (°)	0.3	0.3	0.15
Resolution range (Å)	47.78-1.7(1.79-1.7)	47.74-1.85 (1.94-1.85)	47.64-1.60 (1.63-1.60)
Space group	$P4_12_12$	$P4_12_12$	$P4_12_12$
Unit cell<br>a,b,c (Å)<br>α, β, γ (°)	 141.03, 141.03, 95.56<br>90, 90, 90	 141.05, 141.05, 95.48<br>90, 90, 90	 140.44, 140.44, 95.28<br>90, 90, 90
Crystal mosaicity (°)	0.13	0.115	0.097
No. of molecules in AU	2	2	2
Wilson B-factor	23.4	25.8	21.1
Total reflections	2811648	2170478	4163950
Unique reflections	105810	82374	125165
Multiplicity	26.6 (25.4)	26.4 (26.2)	33.26 (33.48)
Completeness (%)	100 (100)	100 (100)	100 (100)
Mean I/sigma(I)	18.5 (2.9)	15.4 (2.9)	25.13 (3.7)
R_{sigma} (%)	3.1 (36.1)	3.9 (36.2)	2.3 (27.9)
R_{int} (%)	13.67 (77)	15.14 (81)	10.38 (74)
**Refinement**			
Resolution range (Å)	45.25-1.7	45.22-1.85	45.11-1.60
Reflections used in refinement	105721	82292	125077
Reflections used for R-free	5281	4123	6292
R_{work} (%)	14.71	15.59	15.2
R_{free} (%)	16.5	17.99	16.8
**Stereochemical parameters**			
Number of non-hydrogen atoms<br>Macromolecules<br>Ligands<br>Water	5647<br>613<br>9<br>618	5429<br>612<br>11<br>393	5729<br>614<br>10<br>618
R.m.s deviations<br>Bond length (Å)<br>Bond angles (°)	 0.007<br>1.098	 0.007<br>1.103	 0.006<br>1.161
Ramachandran plot<br>Favored (%)<br>Additionally allowed (%)<br>Outliers (%)	 99.18<br>0.82<br>0	 99.51<br>0.49<br>0	 99.18<br>0.82<br>0
B-factors (Å^2)	30.00	33.00	29.00
PDB ID	7O9J	7O9L	7ODZ

R_{free} is calculated for a randomly chosen 5 % subset of reflections.
